# Discharge to inpatient rehabilitation following arthroplasty is a strong predictor of persistent opioid use 90 days after surgery: a prospective, observational study

**DOI:** 10.1186/s12891-023-06142-1

**Published:** 2023-01-14

**Authors:** Deanne E. Jenkin, Ian A. Harris, Joseph Descallar, Justine M. Naylor

**Affiliations:** 1 South West Sydney Clinical School, University of NSW, Sydney, Australia; 2 Whitlam Orthopaedic Research Centre, Liverpool, Australia; 3 Ingham Institute of Applied Medical Research, Liverpool, Australia; 4 South West Sydney Local Health District, Liverpool, Australia

**Keywords:** Opioid, Pain, Analgesics, Arthroplasty, Joint surgery, Rehabilitation, In-patient rehabilitation

## Abstract

**Background:**

Total knee and hip arthroplasty are considered a clinically and cost-effective intervention, however, persistent pain post-surgery can occur, and some continue to take opioid medications long-term. One factor which has infrequently been included in prediction modelling is rehabilitation pathway, in particular, one which includes inpatient rehabilitation. As discharge to inpatient rehabilitation post-arthroplasty is common practice, we aimed to identify whether rehabilitation pathway (discharge to in-patient rehabilitation or not) predicts continued use of opioids at 3 months (90 days) post- total knee arthroplasty (TKA) and total hip arthroplasty (THA) whilst controlling for other covariates.

**Methods:**

The study was nested within a prospective observational study capturing pre-operative, acute care and longer-term data from 1900 osteoarthritis (OA) patients who underwent primary TKA or THA. The larger study involved a part-random, part-convenience sample of 19 high-volume hospitals across Australia. Records with complete pre-and post-operative analgesic (35 days and 90 days) use were identified [1771 records (93% of sample)] and included in logistic regression analyses.

**Results:**

Three hundred and thirteen people (17.8%) reported ongoing opioid use at 90 days post-operatively. In the adjusted model, admission to inpatient rehabilitation after surgery was identified as an independent and significant predictor of opioid use at 90-days. Inpatient rehabilitation was associated with almost twice the odds of persistent opioid use at 90-days compared to discharge directly home (OR = 1.9 (1.4, 2.5), *p* < .001).

**Conclusion:**

The inpatient rehabilitation pathway is a strong predictor of longer-term opioid use (90 days) post-arthroplasty, accounting for many known and possible confounders of use including sex, age, insurance status, major complications, smoking status and baseline body pain levels.

**Trial registration:**

The study was nested within a prospective cohort observational study capturing pre-operative, acute-care and longer-term data from patients undergoing primary TKA or THA for osteoarthritis (ClinicalTrials.gov NCT01899443).

## Background

Osteoarthritis (OA) is a chronic and progressive condition affecting joints such as the hip and knee [1]. It is the most common form of arthritis and the predominant condition leading to total knee arthroplasty (TKA) and total hip arthroplasty (THA) [1]. THA and TKA are commonly performed procedures, by way of example, more than 1 million total joint arthroplasties (TJA) are performed annually in the US [2] and are recommended treatment for patients with end-stage OA whereby non-operative treatments have failed [3]. Though arthroplasty is considered a clinically and cost-effective intervention [4], persistent pain post-surgery does occur in approximately 20% of patients [5] and up to 25% of patients continue to take opioid medications long-term after this type of surgery [6, 7].

The best available evidence does not support opioid analgesia for the management of OA due to increased risk of adverse events, [8] yet opioid use is common in patients prior to arthroplasty [9]. A recent meta-analysis estimated about 24% of patients awaiting TKA or THA were chronic opioid users [10]. Further, studies have suggested that opioid use pre-operatively and during the acute hospital experience is associated with more complicated and painful recoveries with increased likelihood of chronic opioid use post-operatively [11–14]. One factor which has infrequently been included in prediction modelling is rehabilitation pathway while opioid analgesics use during inpatient rehabilitation is considered essential to reduce pain and improve function during treatment interventions [15]. It is possible then that more prolonged exposure to opioids during inpatient rehabilitation may lead to increased reliance on opioids post discharge. As discharge to inpatient rehabilitation post-arthroplasty is common practice in many countries, [16–18] a link with ongoing opioid use would be concerning.

There is interest in investigating opioid use and related harms in pre- and post-operative populations, however, currently available evidence is often retrospective, lacking in patient reported outcomes and largely capturing only opioid utilisation via administrative datasets as opposed to patient-reported opioid consumption [19]. Identification of those at risk and understanding characteristics predictive of longer-term opioid use post-operatively could allow reduction in harm via multiple targeted pathways. This secondary analysis of prospectively collected data aimed to identify whether rehabilitation pathway (discharge to in-patient rehabilitation or not) predicts continued use of opioids at 3 months (90 days) post-surgery whilst controlling for other covariates.

## Patients and methods

### Study design and participants

The study was nested within a prospective cohort study capturing pre-operative, acute-care and longer-term data from patients undergoing primary TKA or THA for osteoarthritis with full methods described elsewhere [20]. The larger study involved a part-random, part-convenience sample of 19 high-volume sites (defined as performing over 275 knee or hip arthroplasties annually) from five Australian States. The inclusion criteria for participants in the study were: consenting adults (over 18 years) with a primary diagnosis of osteoarthritis undergoing primary TKR or THR; sufficient English to comprehend the protocol; and available to participate in follow-up for 12 months.

### Ethical approval and consent to participate

The study was approved by the following Human Research Ethics Committees (HREC) prior to study commencement: Hunter New England HREC (NSW); St Vincent’s Health and Aged Care HREC (Queensland); Austin Health HREC (Victoria); Barwon Health HREC (Victoria); Epworth HREC (Victoria); Calvary Health Care Clinical and Research Ethics Committee (Tasmania) and; Calvary Healthcare Adelaide HREC (South Australia). Prior to commencement the study protocol was registered on the 15 July 2013 (NCT01899443) [21]. All participants provided written, informed consent and all methods were performed in accordance with the relevant guidelines and regulations.

### Baseline data and post-operative outcomes

A consecutive series of eligible people who provided informed consent to participate in this prospective observational study provided basic demographic, sociodemographic, comorbid and analgesia usage data during a pre-admission visit or telephone call 2–6 weeks prior to surgery. Pre-operative opioid use for any reason was also obtained from the medical record. Participants also completed patient-reported outcomes measures (PROMs). The ASA classification of physical health was used to grade comorbid pre-operative health, the ASA score is a subjective assessment of overall health based on five classes (I. healthy fit patient; II. mild systemic disease; III. severe systemic disease that is not incapacitating; IV incapacitating disease with constant threat to life; V. moribund, not expected to live 24 h with or without surgery) [22]. Acute-care outcomes such as length of stay and inpatient rehabilitation were provided by the sites using a standardised pro-forma and verified by site audit at a later time. Inpatient rehabilitation includes physiotherapy, occupational therapy, and speech/language therapy, delivered under the supervision of professionally qualified allied health staff. Inpatients receive a minimum of three hours per day for who have the capacity to tolerate this amount of therapy which should occur on a minimum of five days per week [23]. Participant ineligibility included revision surgery, documented dementia, rheumatoid arthritis in the joint being replaced and another arthroplasty planned within three months of the index surgery.

Thirty-five- and ninety-day post-operative opioid use and PROM data were obtained via telephone follow-up by trained study personnel not involved in care delivery. The Oxford Knee or Hip Score (OKS, OHS) [24] and the EuroQol survey (EQ-5D-5L) [25] were used to measure joint-specific impairment and health-related quality of life, respectively. The OKS and OHS comprise 12 joint-specific Likert-style questions each concerning pain and functional impairment over the last four weeks. Each item is scored from zero (maximal discomfort/pain or maximal impairment) to four (no pain/discomfort or impairment), providing a total score out of 48 with higher scores indicating better joint status.

The EuroQol (EQ-5D-5L) provides a simple descriptive profile in five health dimensions (mobility, self-care, usual activities, pain/discomfort, and anxiety/depression) and a single index value for health status. Each question is scored as a single number 1 (no problems), 2 (some problems) 3 (moderate problems), 4 (severe problems), 5 (extreme problems). A 0–100 VAS scale for perceived overall health is also part of the tool: 0 indicating worst health, and 100 indicating best health imaginable.

### Data definitions

An opioid analgesic was defined as codeine 15 mg or over, buprenorphine, dextropropoxyphene, fentanyl, hydromorphone, methadone, oxycodone, or tramadol. A comorbidity was defined as the presence of one or more additional disorders including heart disease, stroke, high blood pressure, high cholesterol, cardiac disease, kidney disease, liver disease, diabetes, cancer, lung disease, depression, anxiety or mental health disease, bleeding disorders, gastroesophageal reflux disease, sleep apnoea, neurological disease, parkinson’s disease, co-occurring with the primary disease (OA), diagnosed by a doctor and requiring daily medications. The presence of any comorbid condition was treated as binary (Yes/No).

A major complication was defined as experiencing at least one of any of the following events: pulmonary emboli, deep venous thrombosis, deep surgical site infection, revision, reoperation, joint fracture, dislocation, manipulation under anaesthesia, stroke, acute myocardial infarction and/or re-admission. Major complications were grouped, based on time of occurrence, as acute (occurring during hospital admission) or post-discharge (occurring after discharge up until 90 days post-surgery).

### Statistical analysis

Data were analysed using SAS Enterprise Guide version 8.2. Baseline characteristics were compared for those taking opioids at 90 days post-operatively (the opioid group) and those not taking opioids (the non-opioid group). The relationship between collected or measured variables and opioid grouping were assessed in univariate logistic regression. The exposure of interest was inpatient rehabilitation or not, while all other variables were chosen as being known or suspected predictors of ongoing opioid use. A backward-elimination model was utilised which started with all explanatory variables and then discarded the least statistically significant variables, one by one. The final model was chosen when each variable remaining in the equation was statistically significant. Some variables were collapsed prior to their addition into the model, specifically, education and smoking status. Interactions between inpatient rehabilitation with acute- major complications, and inpatient rehabilitation with post-major complications were considered in the models. A sensitivity analysis was performed for the opioid naïve patients (non-opioid users pre-operatively). Sample size was dictated by the original study hence no a priori calculation was performed. Odds ratios (ORs) were calculated with the 95% confidence interval (95% CI). A *p*-value < 0.05 was considered significant. Patients with any missing predictor data were removed from analysis.

## Results

**Fig. 1:**
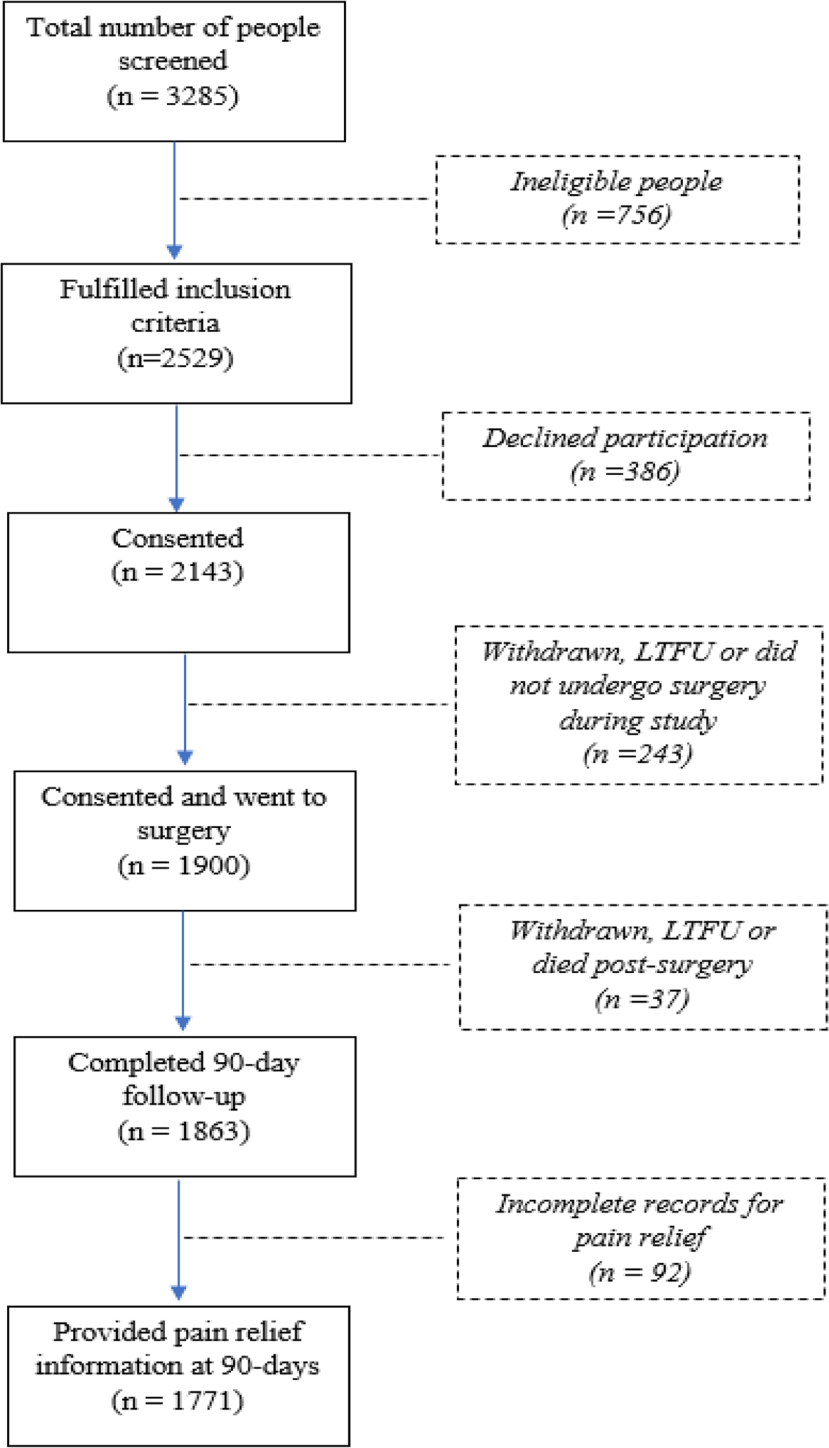
Flow diagram of study

A consecutive sample of 1900 patients diagnosed with OA consented and underwent surgery of total hip or knee arthroplasty from 14 August 2013 to 22 January 2015 (Fig. 1). At 90 days, 1863 people completed follow-up at 90-days (98.1%) post-surgery. Records with complete pre-and post-operative analgesic use were identified and included in analyses (1771 out of 1900 total records). Three hundred and ten people (17.5%) reported using opioids to manage their musculoskeletal pain at initial assessment, including 188 (60.6%) women and 122 (39.4%) men (Table 1). Three hundred and thirteen people (17.8%) reported ongoing opioid use at 90 days post-operatively. Univariate logistic regression was conducted on 18 explanatory variables prior to inclusion into the backwards regression model (Table 2). Sixty-one patients (3.4%) had at least one missing predictor information. Among the opioid users at 90 days, 62.3% were opioid naïve prior to surgery.

**Table 1:** Descriptive characteristics of the total hip and total knee arthroplasty study cohort

Characteristic	All	Pre-operative opioid user	Post-op 35 days opioid user	Post-op 90 days opioid user
**n** (%)	1771 (100)	310 (17.5)	735 (41.5)	313 (17.7)
**Age**, *mean (SD)*	67.3 (9.7)	65.4 (9.9)	67.2 (9.4)	65.9 (9.2)
**Sex**, *female*	953 (53.8)	188 (60.6)	437 (59.5)	202 (64.5)
**Hospital,** *Private*	928 (52.4)	125 (40.3)	372 (50.6)	153 (48.9)
**Surgery Joint**				
Knee	1017 (57.4)	165 (53.2)	524 (71.3)	237 (75.7)
Hip	754 (42.6)	145 (46.8)	211 (28.7)	76 (24.3)
Bilateral	88 (5.0)	9 (2.9)	53 (7.2)	22 (7.0)
**Previous TKA**	473 (26.7)	52 (16.8)	117 (15.9)	49 (15.7)
**Previous THA**	292 (16.5)	89 (28.7)	181 (24.6)	73 (23.3)
**BMI,** *mean (SD)*	31.0 (6.4)	32.2 (7.0)	31.7 (6.7)	32.6 (7.2)
**Education (highest level)**				
Year 10 or below	656 (37.0)	122 (39.4)	283 (38.5)	202 (64.5)
Senior Secondary or above	1115 (63.0)	188 (60.7)	441 (60.0)	192 (61.3)
**Comorbid condition**	1605 (90.6)	285 (91.9)	686 (93.3)	301 (96.2)
**Acute major complication**	59 (3.3)	9 (2.9)	40 (5.4)	59 (18.8)
**Post major complication**	135 (7.6)	29 (9.4)	93 (12.7)	135 (43.1)
**Tobacco use**				
Current smoker	152 (8.6)	41 (13.2)	83 (11.3)	42 (13.4)
Former smoker	735 (41.5)	128 (41.3)	302 (27.6)	129 (41.2)
**Length of Stay**, *mean (SD)*	5.6 (2.5)	5.5 (2.5)	6.0 (2.6)	6.5 (3.2)
**Discharge Destination**				
Inpatient Rehabilitation	523 (29.5)	77 (24.8)	274 (37.3)	127 (40.6)
**Employed at time of surgery**	492 (27.8)	83 (26.8)	185 (25.2)	78 (24.9)
**EQ-5D Baseline**				
** Mobility**				
No problems	129 (7.3)	7 (2.3)	40 (5.4)	9 (2.9)
Slight problems	375 (21.2)	41 (13.2)	142 (19.3)	64 (20.4)
Moderate problems	776 (43.8)	122 (39.4)	331 (45.3)	135 (43.1)
Severe problems	460 (26.0)	133 (42.9)	209 (28.4)	96 (30.7)
Unable	15 (0.84)	6 (1.9)	8 (1.1)	6 (1.9)
** Self-care**				
No problems	952 (53.8)	122 (39.4)	407 (55.4)	163 (52.1)
Slight problems	441 (24.9)	91 (29.4)	172 (23.4)	77 (24.6)
Moderate problems	672 (37.9)	73 (23.5)	110 (15.0)	54 (17.3)
Severe problems	295 (14.6)	21 (6.8)	38 (5.17)	15 (4.8)
Unable	83 (4.7)	2 (0.7)	1 (0.1)	1 (0.3)
** Activities**				
No problems	252 (14.2)	22 (7.1)	107 (14.6)	34 (10.8)
Slight problems	453 (25.6)	63 (20.3)	177 (24.1)	80 (25.6)
Moderate problems	672 (37.9)	117 (37.7)	272 (37.0)	114 (36.4)
Severe problems	295 (16.7)	80 (25.8)	132 (18.0)	62 (19.8)
Unable	83 (46.8)	27 (8.7)	42 (5.7)	20 (6.4)
** Pain/Discomfort**				
No pain or discomfort	40 (2.2)	1 (0.3)	10 (1.4)	2 (0.6)
Slight pain or discomfort	279 (15.8)	18 (5.8)	103 (14.0)	31 (9.9)
Moderate pain or discomfort	826 (46.6)	118 (38.1)	337 (45.9)	131 (41.9)
Severe pain or discomfort	513 (29.0)	134 (43.2)	237 (32.2)	116 (37.1)
Extreme pain or discomfort	97 (5.4)	38 (12.3)	43 (5.8)	29 (9.3)
** Depression/Anxiety**				
Not anxious/depressed	965 (54.5)	126 (40.6)	366 (49.8)	150 (47.9)
Slightly anxious/depressed	474 (26.8)	97 (31.3)	204 (27.8)	31 (9.9)
Moderately anxious /depressed	230 (13.0)	63 (20.3)	113 (15.4)	58 (18.5)
Severely anxious or depressed	64 (3.6)	15 (4.8)	34 (4.6)	13 (4.2)
Extremely anxious or depressed	18 (1.0)	8 (2.6)	8 (1.1)	7 (2.2)
**Health Status VAS**, *mean (SD)*	70.9 (18.6)	64.0 (20.2)	69.2 (18.7)	66.4 (19.2)
**Baseline Oxford Score**, *mean (SD)*	21.2 (8.6)	16.5 (20.2)	20.0 (8.3)	18.7 (8.2)

*Legend*: Number and percentage shown unless otherwise stated; Hospital = private or public hospital; Total Knee Arthroplasty; THA- Total Hip Arthroplasty; Baseline measure = pre-operative measure; EQ-5D: EuroQol 5 dimensions; Health Status VAS- EQ-5D Visual analogue scale (VAS) (100 = the best health you can imagine); Oxford Knee or Hip Score (tool range 0-48, higher scores are better).

**Table 2:** Cohort characteristics stratified by 90-day opioid use post-surgery

Characteristic	90-day non-opioid user	90-day opioid user	Odds Ratio	95% Confidence Interval		*P*-value*
**n** (%)	1458 (82.3)	313 (17.7)				
**Age**, *mean (SD)*	67.6 (9.8)	65.9 (9.2)	.982	.97	.994	0.004
**Sex**, *female*^+^	751 (51.5)	202 (64.5)	.584	.453	.752	< 0.001
**Pre-op opioid user**	192 (13.2)	118 (37.7)	3.99	3.033	5.25	< 0.001
**Hospital,** *Private*^+^	775 (53.2)	153 (48.8)	.843	.66	1.076	0.17
**Surgery Joint**						
Knee	780 (53.5)	237 (75.7)	2.711	2.052	3.58	< 0.001
**Bilateral Hip or Knee**	66 (4.5)	22 (7.0)	1.595	.968	2.626	0.067
**Previous THA/ TKA**	400 (27.4)	73 (23.3)	.804	.604	1.07	0.135
**Education,** *Year 10 or below*^+^						
Completed Year 11 or above	923 (63.3)	192 (61.3)	.93	0.722	1.198	0.574
**Comorbid condition**	1304 (89.4)	301 (96.2)	2.961	1.624	5.398	< 0.001
**Acute major complication**	1419 (97.3)	293 (93.6)	2.484	1.428	4.32	0.001
**Post-major complication**	1374 (94.2)	262 (83.7)	3.184	2.195	4.62	< 0.001
**Pre-op opioid use**	192 (13.2)	118 (37.7)	3.99	3.033	5.25	< 0.001
**Tobacco use**						
Current smoker	110 (7.5)	42 (13.4)	1.903	1.303	2.78	< 0.001
**Inpatient Rehabilitation**	396 (27.2)	127 (40.6)	1.831	1.421	2.359	< 0.001
**Employed at time of surgery**	413 (28.3)	79 (25.2)	0.866	0.655	1.146	0.315
**EQ-5D Baseline**						
**Pain/Discomfort**			1.55^†^	1.335	1.799	< 0.0001
No pain or discomfort	38 (2.6)	2 (0.6)				
Slight pain or discomfort	248 (17.0)	31 (9.9)				
Moderate pain or discomfort	694 (47.6)	132 (42.2)				
Severe pain or discomfort	395 (27.1)	118 (37.7)				
Extreme pain or discomfort	68 (4.7)	29 (9.3)				
**Baseline Oxford Knee or Hip Score,** *mean (SD)*	21.8 (8.6)	18.7 (8.1)	.957	.943	.972	< 0.0001
**ASA**, 3 +	432 (30.3)	131 (43.2)	1.725	1.337	2.225	< 0.0001

*Legend*: Number and percentage shown unless otherwise stated; Reference = No, unless otherwise indicated; SD = standard deviation; Education = highest completed level; Year 11 or above = senior secondary high school

^+^Reference group

^*^p-values based on univariate logistic regression

^†^Odds Ratio is per increase in pain/discomfort level

In the adjusted model, admission to inpatient rehabilitation after surgery was identified as an independent and significant predictor of opioid use at 90 days and was associated with almost twice the odds of persistent opioid use at 90 days than discharge directly (OR = 1.9, 95% Confidence Interval (1.4, 2.5)). There were no significant interaction terms in the final model (Table 3). A sensitivity analysis was performed for the opioid naïve which found minimal difference to all variables with the exception of having a co-morbid condition which becomes non-significant. The main result that inpatient rehab predicts 90-day opioid use remains (OR = 2.02, 95% CI (1.44, 2.84), *p* < 0.0001).

**Table 3:** Adjusted model exploring discharge destination as a predictor of opioid use at 90-days post-operatively

Characteristic	Odds Ratio	95% Confidence Interval		*p*-value
**Age** (*per year)*	0.972	0.958	0.987	0.0003
**Sex** *(reference female)*	0.69	0.517	0.921	0.0118
**Surgery joint** *(reference THA)*	3.128	2.268	4.315	< .0001
**Co-morbid condition**	2.174	1.104	4.282	0.0248
**Major complication post-discharge**	2.863	1.871	4.381	< .0001
**Pre-operative opioid use**	3.943	2.849	5.459	< .0001
**Current smoker at time of surgery**	1.976	1.265	3.087	0.0028
**Inpatient Rehabilitation**	1.876	1.394	2.523	< .0001
**EQ-5D Baseline Pain/Discomfort** *(per increase in pain/discomfort level****)***	1.261	1.06	1.501	0.009
**ASA score** *(reference* = *1,2)*	1.343	1.003	1.799	0.0477

*Legend*: Reference group = No, unless otherwise stated; Co-morbid condition: The presence of any medicated comorbid condition was treated as binary (Yes/No). ASA score: The American Society of Anesthesiologists (ASA) score

## Discussion

Recently, opioid use has seen much attention globally due to the rise in acute harms from overuse/misuse resulting in a large burden of disease and mortality [26, 27]. Numerous studies have identified factors predictive of longer-term opioid use, to identify those at risk and minimise harm [19, 28, 29]. However, the inpatient rehabilitation pathway has been largely overlooked as a potential risk factor for ongoing opioid use yet is it a common pathway after hip or knee arthroplasty. In this large, prospective cohort, whilst accounting for many important covariates including the occurrence of a complication acutely or post-discharge, discharge to inpatient rehabilitation after hip or knee arthroplasty was identified as an independent and significant predictor of opioid use at 90-days. Additionally, discharge to inpatient rehabilitation was independently associated with ongoing opioid use regardless of whether patients are taking opioids pre-operatively or not. In general terms, this study illustrates that opioid use is common across all aspects of the patient experience encompassing pre-operative, acute and post-operative care, and this is independent of insurance status (public versus private).

While other research has identified risk factors for longer-term opioid use, they have often included small cohorts, retrospective collected information or administrative data to identify use. Our data are in line with a previous retrospective study conducted in the US [19], and more recently, in line with data obtained from a randomised trial demonstrating that the risk of purchasing opioids after discharge from hospital was almost double for people randomised to 10-days of inpatient rehabilitation compared to those discharged directly home (relative risk 1.8 95% 1.2 to 2.8) [30]. While pain management following TKA and THA is complex, our study does not provide any insights regarding why discharge to inpatient rehabilitation is strongly predictive of ongoing opioid use. We may hypothesise that it is either prolonged exposure to opioids whilst in the inpatient facility that increases a person’s reliance on them after discharge, or ongoing opioid prescriptions may be provided at discharge from the facility.

Utilisation of inpatient rehabilitation post-arthroplasty is common practice in many countries, making the independent link found for persistent opioid use of clinical concern. Further, a recent large US retrospective cohort study found that disadvantaged people are more likely to be discharged to an inpatient rehabilitation facility [31]—another concerning reality in light of the fact that abuse of prescription opioids are associated with younger age, poverty, and unemployment [32]. Another Canadian study found that, among patients who were admitted to inpatient rehabilitation for various musculoskeletal conditions—largely for factures, trauma, and arthroplasty—and who were opioid naïve, and had no history of chronic pain prior, the majority (63%) were prescribed opioids at discharge [14]. In general, between 21 and 43% of individuals that take prescriptions opioids for chronic musculoskeletal pain may misuse them or develop substance abuse disorders while our small single-centre RCT highlights that it may be possible to provide similar pain relief using a milder-opioid alternative after other types of musculoskeletal surgery [33–36]. This study highlights that inpatient rehabilitation providers need to be aware of their role in contributing to potentially inappropriate use of opioids. Understanding drivers of persistent use post-surgery can help inform targeted strategies such as routinely incorporating weaning strategies or reducing or ceasing strong opioid prescription at the time of discharge, intended to reduce inappropriate opioid use amongst surgical patients at a time when joint pain should be resolving.

Some limitations of our work require emphasis. Due to the research design (observational study with no comparison groups, random assignment, or establishment of time order) no causal relationships can be inferred though, as stated above, the data align with observations from a randomised study demonstrating a causal relationship. Some potential confounders such as, substance use disorder and variability of prescribing patterns across centres were unable to be accessed hence not included here. Further, beyond the presence of a comorbid condition, type and severity of comorbidity were ignored due to the volume of comorbidities, thus, we have not explored the association between individual comorbidities and persistent opioid use. Finally, we relied on patient self-report for opioid use at 90 days. Whilst this is arguably better than reliance on prescription-based databases where it is known that patients have been prescribed opioids, but unknown if opioids were actually being used, our ‘use’ data have not been validated by objective means.

## Conclusion

The inpatient rehabilitation pathway is a strong predictor of longer-term opioid use (90 days) post-arthroplasty, accounting for many known and possible confounders of use including sex, age, insurance status, major complications, smoking status, and baseline body pain levels. Inpatient rehabilitation providers need to be cognizant of their role in contributing to potentially inappropriate use of opioids post-surgery and routinely incorporate weaning strategies at the time of discharge.

## Data Availability

The datasets used and/or analysed during the current study are available from the corresponding author on reasonable request.

## Abbreviations

ASA: American Society of Anaesthesiologists’ (ASA) classification of physical health
EQ-5D-5L: Euroqol survey instrument
PROMs: Patient-reported outcomes measures
THA: Total hip arthroplasty
TKA: Total knee arthroplasty
OA: Osteoarthritis
OHS: Oxford Hip Score
OKS: Oxford Knee Score

## References

[1] Australian Institute of Health and Welfare (AIHW). Osteoarthritis snapshot. Web report. Cat No: PHE 232. 24 July 2018. https://www.aihw.gov.au/reports/chronic-musculoskeletal-conditions/osteoarthritis/contents/what-is-osteoarthritis

[2] Etkin CD, Springer BD (2017). The American Joint Replacement Registry-the first 5 years. Arthroplasty Today.

[3] Yusuf E (2016). Pharmacologic and Non-Pharmacologic Treatment of Osteoarthritis. Curr Treat Options in Rheum.

[4] Higashi H, Barendregt JJ (2011). Cost-Effectiveness of Total Hip and Knee Replacements for the Australian Population with Osteoarthritis: Discrete-Event Simulation Model. PLoS ONE.

[5] Corke P (2013). Postoperative pain management. Aust Prescr.

[6] Singh JA, Lewallen D (2010). Predictors of pain and use of pain medications following primary Total Hip Arthroplasty (THA): 5,707 THAs at 2-years and 3,289 THAs at 5-years. BMC Musculoskelet Disord.

[7] Valdes AM, Gilson AM, Maurer MA (2015). Use of prescription analgesic medication and pain castastrophizing after total joint replacement surgery. Semin Arthritis Rheum.

[8] da Costa BR, Nüesch E, Kasteler R, Husni E, Welch V, Rutjes AW, et al. Oral or transdermal opioids for osteoarthritis of the knee or hip. Cochrane Database Syst Rev. 2014;(9):CD003115. 10.1002/14651858.CD003115.pub4. 10.1002/14651858.CD003115.pub4 PMC10993204 25229835

[9] Goplen CM, Verbeek W, Kang SH, Jones CA, Voaklander DC, Churchill TA (2019). Preoperative opioid use is associated with worse patient outcomes after total joint arthroplasty: a systematic review and meta-analysis. BMC Musculoskelet Disord.

[10] Zywiel MG, Stroh DA, Lee SY, Bonutti PM, Mont MA (2011). Chronic opioid use prior to total knee arthroplasty. J Bone Joint Surg Am.

[11] Zarling BJ, Yokhana SS, Herzog DT, Markel DCJ (2016). Preoperative and Postoperative Opiate Use by the Arthroplasty Patient. J Arthroplasty.

[12] Inacio MCS, Hansen C, Pratt NL (2016). Risk factors for persistent and new chronic opioid use in patients undergoing total hip arthroplasty: a retrospective cohort study. BMJ Open.

[13] Naylor JM, Pavlovic N, Farrugia M (2020). Associations between pre-surgical daily opioid use and short-term outcomes following knee or hip arthroplasty: a prospective, exploratory cohort study. BMC Musculoskelet Disord.

[14] Furlan AD, Hassan S, Famiyeh IM, Wang W, Dhanju J (2016). Long-term opioid use after discharge from inpatient musculoskeletal rehabilitation. J Rehabil Med.

[15] Naylor JM, Hart A, Harris IA, Lewin AM (2019). Variation in rehabilitation setting after uncomplicated total knee or hip arthroplasty: a call for evidence-based guidelines. BMC Musculoskelet Disord.

[16] Mehta B, Singh JA, Ho K, Nelson C, D'Angelo D, Ibrahim SA (2019). Race, discharge disposition, and readmissions after elective hip replacement: analysis of a large regional dataset. Health Equity.

[17] Benz T, Angst F, Oesch P (2015). Comparison of patients in three different rehabilitation settings after knee or hip arthroplasty: a natural observational, prospective study. BMC Musculoskelet Disord.

[18] Tarity TD, Swall MM (2017). Current Trends in Discharge Disposition and Post-discharge Care After Total Joint Arthroplasty. Curr Rev Musculoskelet Med.

[19] Kim SC, Choudhry N, Franklin JM, Bykov K, Eikermann M, Lii J, Fischer MA, Bateman BT (2017). Patterns and predictors of persistent opioid use following hip or knee arthroplasty. Osteoarthritis Cartilage.

[20] Badge HM, Churches T, Naylor JM, Xuan W, Armstrong E, Gray L, Fletcher J, Gosbell I, Lin C, Harris IA (2021). Non-compliance with clinical guidelines increases the risk of complications after primary total hip and knee joint replacement surgery. PLoS One.

[21] Clinicaltrials.gov [Internet]. Clinical trial registration Bethesda (MD): National Library of Medicine (US); 2013 [30/06/2019]. Available from: https://www.clinicaltrials.gov/ct2/show/NCT01899443?term=Improving+Services+and+Outcomes+for+Joint+Replacement+Patients&draw=2&rank=1

[22] Abouleish AE, Leib ML, Cohen NH. ASA provides examples to each ASA physical status class. ASA Monitor 2015; 79:38–39. http://monitor.pubs.asahq.org/article.aspx?aeticleid=2434536

[23] The Royal Australasian College of Physicians. Standards for Rehabilitation Services. https://www.racp.edu.au/fellows/resources/rehabilitation-medicine-resources/standards-for-rehabilitation-services/staffing. Accessed 13 Jan 2022

[24] Murray DW, Fitzpatrick R, Rogers K (2007). The use of the Oxford hip and knee scores. J Bone Joint Surg Br.

[25] Chatterji R, Naylor JM, Harris IA, Descallar J (2017). An equivalence study: are patient-completed and telephone interview equivalent modes of administration for the EuroQol survey?. Health Qual Life Outcomes.

[26] Manchikanti L, Helm S 2nd, Fellows B, et al. Opioid epidemic in the United States. Pain Physician. 2012;15[3 Suppl]:ES9–38. 22786464

[27] Rudd RA, Aleshire N, Zibbell JE, Gladden RM (2016). Increases in drug and opioid overdose deaths–United States, 2000–2014. MMWR Morb Mortal Wkly Rep.

[28] Lawal OD, Gold J, Murthy A (2020). Rate and Risk Factors Associated With Prolonged Opioid Use After Surgery: A Systematic Review and Meta-analysis. JAMA Netw Open.

[29] Goesling J, Moser SE, Zaidi B, Hassett AL, Hilliard P, Hallstrom B (2016). Trends and predictors of opioid use after total knee and total hip arthroplasty. Pain.

[30] Naylor JM, Buhagiar M, Johns N, Penm J, Adie S, Harris IA, Xuan W (2021). Prescription Opioids Higher Among Knee Arthroplasty Recipients Randomized to Inpatient Rehabilitation. ACR Open Rheumatol.

[31] Mehta B, Goodman S, Ho K, Parks M, Ibrahim SA (2021). Community Deprivation Index and Discharge Destination After Elective Hip Replacement. Arthritis Care Res (Hoboken).

[32] Ives TJ, Chelminski PR, Hammett-Stabler CA, Malone RM, Perhac JS, Potisek NM, Shilliday BB, DeWalt DA, Pignone MP (2006). Predictors of opioid misuse in patients with chronic pain: a prospective cohort study. BMC Health Serv Res.

[33] Manchikanti L, Vallejo R, Manchikanti KN, Benyamin RM, Datta S, Christo PJ (2011). Effectiveness of long-term opioid therapy for chronic non-cancer pain. Pain Physician.

[34] Martell BA, O'Connor PG, Kerns RD, Becker WC, Morales KH, Kosten TR (2007). Systematic Review: Opioid Treatment for Chronic Back Pain: Prevalence, Efficacy, and Association with Addiction. Ann Intern Med.

[35] Vowles K, McEntee M, Julnes P, Frohe T (2015). Rates of opioid misuse, abuse, and addiction in chronic pain: a systematic review and data synthesis. Pain.

[36] Jenkin DE, Naylor JM, Descallar J, Harris IA (2021). Effectiveness of Oxycodone Hydrochloride (Strong Opioid) vs Combination Acetaminophen and Codeine (Mild Opioid) for Subacute Pain After Fractures Managed Surgically: A Randomized Clinical Trial. JAMA Netw Open.

